# Endometrial Osseous Metaplasia—A Rare Cause of Infertility with Unknown Etiology

**DOI:** 10.3390/medicina59101803

**Published:** 2023-10-10

**Authors:** Vlad Iustin Tica, Iulia Postolache, Madalina Boșoteanu, Mariana Aschie, Irina Tica, Cristian Ionut Orasanu, Roxana Cleopatra Penciu, Andrei Adrian Tica, Liliana Steriu, Rudy Leon De Wilde, Oana Sorina Tica

**Affiliations:** 1 Department of Obstetrics and Gynecology, “Saint Andrew” University Emergency Hospital Constanta, Faculty of Medicine, Academy of Romanian Scientists University “Ovidius”, 900527 Constanta, Romania; vtica@eeirh.org (V.I.T.); roxanapenciu@yahoo.com (R.C.P.); lilianasteriu@icloud.com (L.S.); 2 Obstetrics and Gynecology, Euromaterna Hospital, 900402 Constanta, Romania; drpostolacheiulia@gmail.com; 3 Department of Pathology of “Saint Andrew” University Emergency Hospital Constanta, Faculty of Medicine, University “Ovidius”, 900527 Constanta, Romania; mbosoteanu@yahoo.com; 4 Center for Research and Development of the Morphological and Genetic Studies of Malignant Pathology, Faculty of Medicine, University “Ovidius”, 900591 Constanta, Romania; aschiemariana@yahoo.com (M.A.); critian.ionut@gmail.com (C.I.O.); 5 Department of Internal Medicine of “Saint Andrew” University Emergency Hospital Constanta, Faculty of Medicine, University “Ovidius”, 900470 Constanta, Romania; 6 Department of Pharmacology, University of Medicine and Pharmacy, 200349 Craiova, Romania; 7 Department of Obstetrics and Gynecology, County Emergency Hospital, 200642 Craiova, Romania; oanabanica25@yahoo.com; 8 “Pius” Hospital Oldenburg, University Hospital for Gynecology, Carl von Ossietzky University Oldenburg, 26129 Oldenburg, Germany; 9 Department of Obstetrics and Gynecology, University of Medicine and Pharmacy, 200642 Craiova, Romania

**Keywords:** endometrial calcification, osseous metaplasia, primary infertility

## Abstract

*Background:* Osseous tissue in the endometrium is a rare find, and it is most often discovered when the patient presents with infertility. It is frequently associated with dysmenorrhea and abnormal menstrual bleedings. Although its etiology remains unclear, in almost all described cases until now, the patient has an obstetrical history. *Case report*: In this report, we present a unique case of endometrial osseous metaplasia in a 27-year-old primary infertile patient. The transvaginal ultrasound revealed a 18/13/7 mm hyperechoic endometrial mass with posterior acoustic shadowing and no flow on color Doppler. A hysteroscopic examination found a polygonal calcification on the endometrial posterior face of the uterine cavity, in the corporeal isthmic region, which was extracted. The histopathological evaluation revealed microscopic elements compatible with endometrial calcification. The patient had a good postoperative course and the complex endocrinologic, immunologic and electrolytical investigation failed to prove any abnormality. Follow-up transvaginal ultrasound examinations revealed no modifications. Three years later, the patient conceived spontaneously, had an uneventful pregnancy and delivered a full-term fetus. *Conclusion*: We assumed that this entity can be a serious cause of infertility since the patient had a long history of (primary) infertility and its resection made the pregnancy’s occurrence possible. Finally, since neither history of abortion or chronic inflammation nor any abnormal laboratory test were noticed, we concluded that the etiology of this entity remained unclear.

## 1. Introduction

Endometrial osseous metaplasia (EOM) is a rare condition, characterized by the presence of mature or immature bone in the endometrium [1,2]. In the literature, this abnormality was also described as endometrial ossification, ectopic intrauterine bone or heterotopic intrauterine bone [3].

Many patients are asymptomatic, with the lesion being discovered in regular gynecological exams or, far more often, during an investigation for infertility [1,4,5,6,7]. There is no specific symptomatology, but EOM could be associated with menstrual irregularities, pelvic pain, dyspareunia, dysmenorrhea and/or recurrent abortions [7].

The etiology in unclear, but almost all cases cited had an obstetrical history [1,7,8,9].

We present a rare case which, beyond the rarity of the endometrial calcification, has the particularity of not being associated with either previous intrauterine maneuvers or with a history of abortion. Furthermore, no previous gynecological or general health problems were revealed. 

Finally, an analysis of its possible etiology is also discussed.

### Case Report

We describe the case of a 27-year-old female patient who presented to our department with a history of primary infertility. She was married for 2 years and never became pregnant. Neither she nor her husband had a significant family or personal history.

She had a 26–28 days regular menstrual cycle, lasting 5 days, without dysmenorrhea or abnormal vaginal discharge. She did, however, have menorrhagia, but the patient was unable to mention when this became more pronounced since her regular blood loss was subjectively increased from her first menstruation, which occurred at 12 years old.

The physical and gynecological examinations were normal.

**Figure 1:**
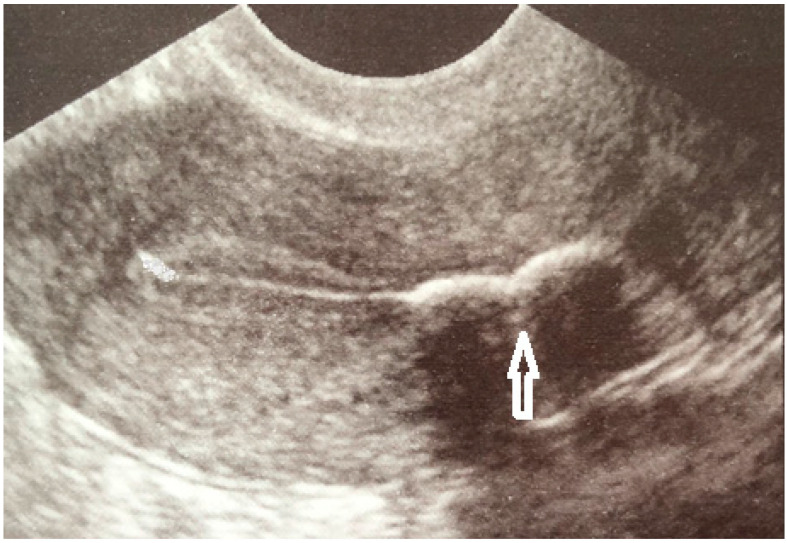
Ultrasound image of intrauterine calcification.

The transvaginal ultrasound revealed a 18/13/7 mm hyperechoic endometrial mass with posterior acoustic shadowing in the isthmic and corporeal region of the posterior uterine wall, with no flow on color Doppler (Figure 1).

**Figure 2:**
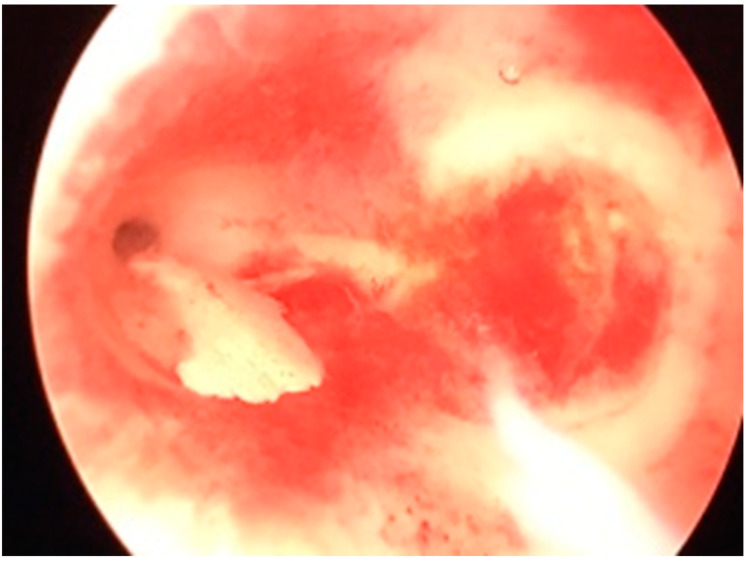
Hysteroscopic view of the uterine cavity, revealing the calcification (panoramic view).

**Figure 3:**
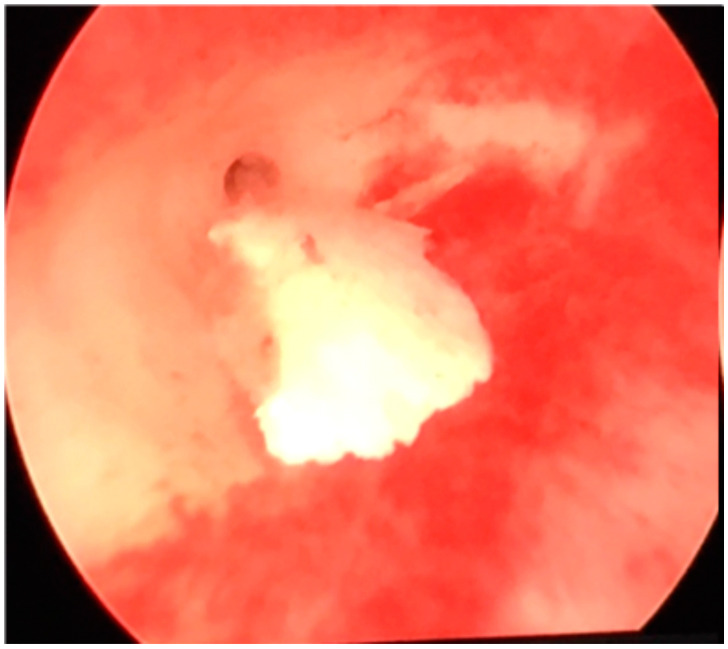
Hysteroscopic view of intrauterine calcification (detail).

A hysteroscopic examination was decided upon and it was performed immediately after the following menstruation. Previously, a complete cervical cytological and microbiological investigation was conducted which proved to be normal. Hysteroscopy revealed a uterine cavity with the endometrium aspect corresponding to the patient’s menstrual phase, with normal-appearing tubal ostia and a normal cervical canal. No sign of endometritis was observed. A polygonal calcification was found on the posterior face of the uterine cavity, located at the right tubal ostium, possibly pushed by the distension media flow (Figure 2 and Figure 3). 

The calcification zone was extracted and sent to pathology. 

**Figure 4:**
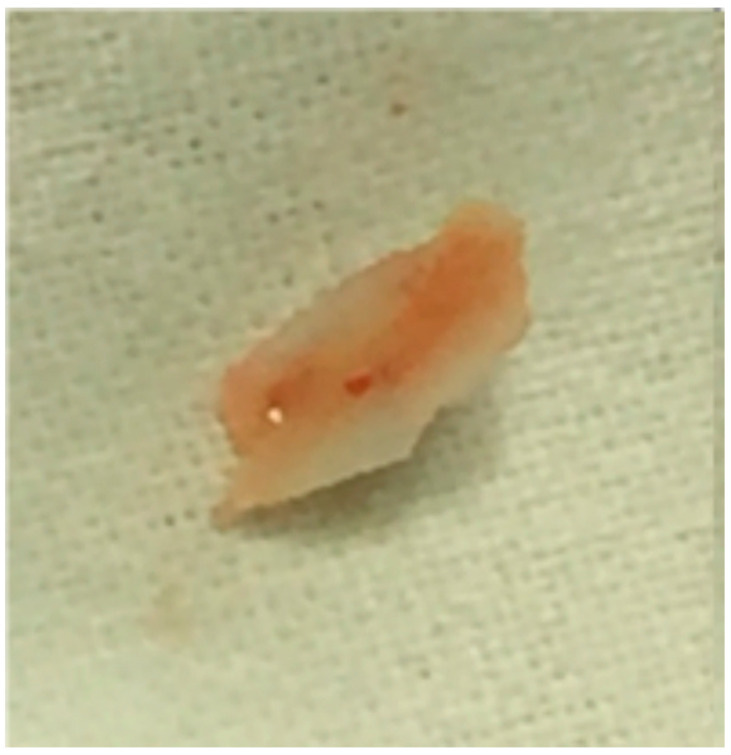
Resected intrauterine calcification.

The biopsy specimen included a yellowish-gray tissue fragment of increased consistency. It measured 13/6/3 mm (Figure 4). 

The patient had a good postoperative course and she was discharged from the hospital the day after the intervention.

**Figure 5:**
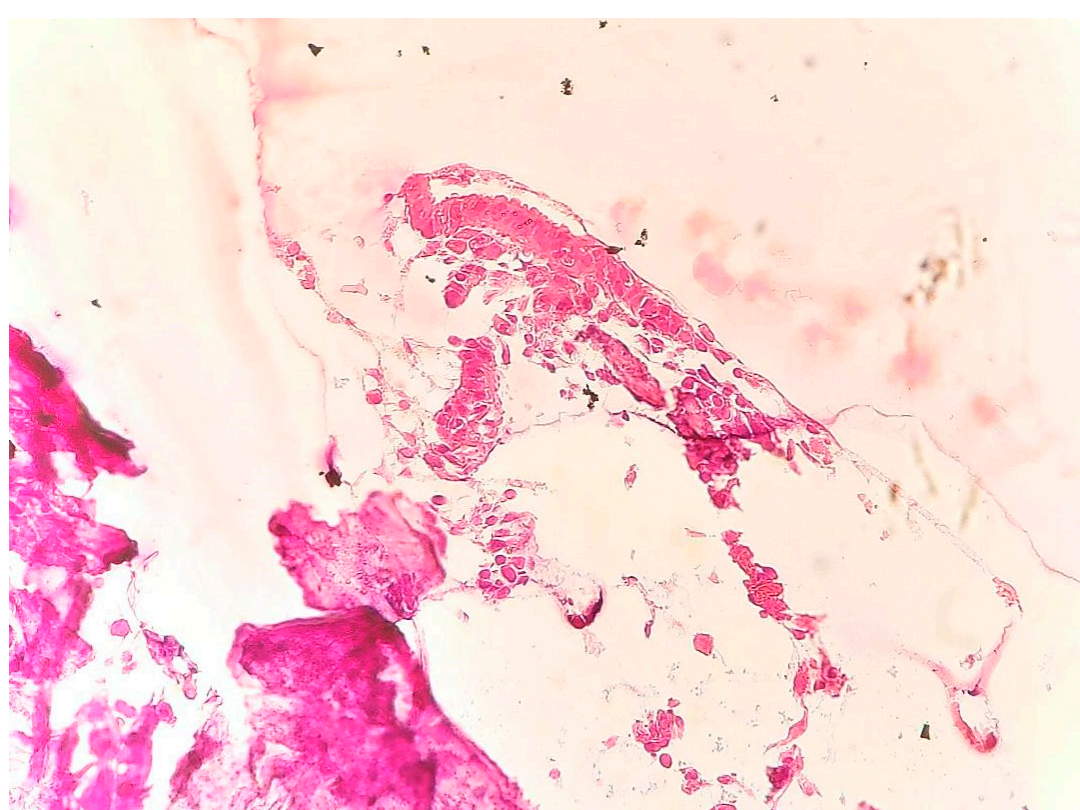
Basophilic acellular material, including glandular epithelial fragments (H.E.; ×40).

**Figure 6:**
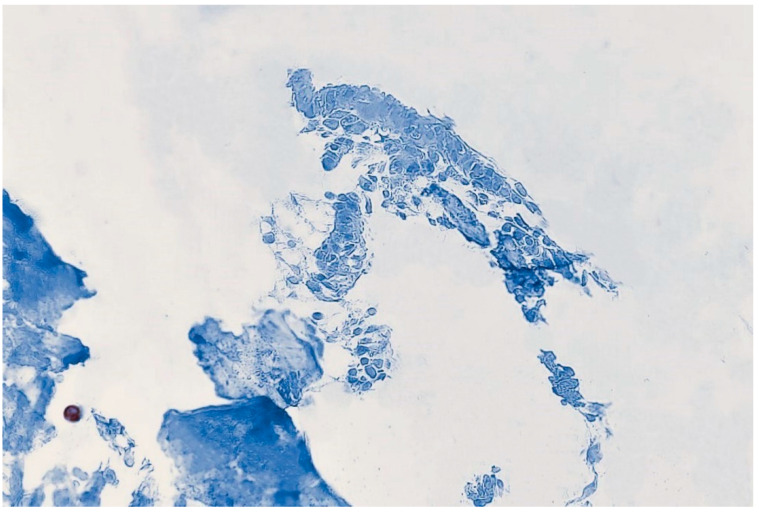
CD138 immunostaining.

The pathological evaluation revealed the presence of a basophilic acellular material compatible with endometrial calcification [10]. It was associated with fragments of glandular epithelium without changes in chronic inflammation (CD138-negative in the underlying stroma and at the epithelial level) and leukocyte infiltrate in reduced quantity (Figure 5 and Figure 6). 

The antigen was negative throughout the epithelial cells, with an intense positive reaction in the control (×40).

**Figure 7:**
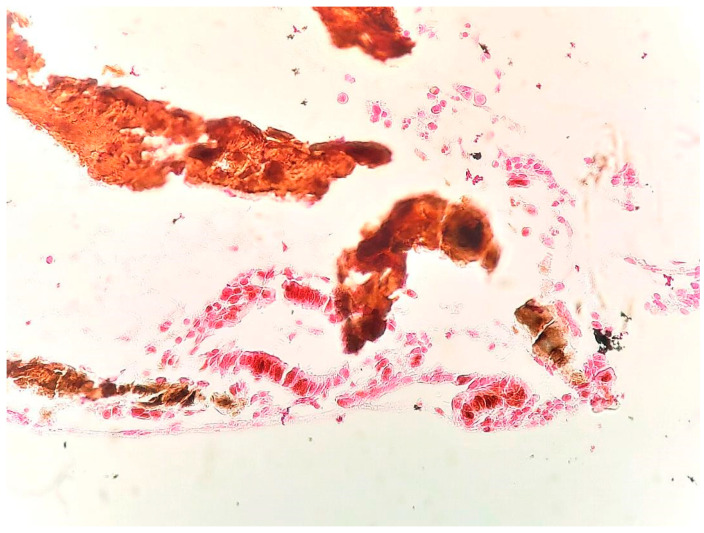
Brown-black aspect of the amorphous deposits (von Kossa ×40).

The use of von Kossa’s special staining confirmed the presence of calcium in the tissue by revealing a brown-black aspect of the amorphous deposits [10] (Figure 7).

**Figure 8:**
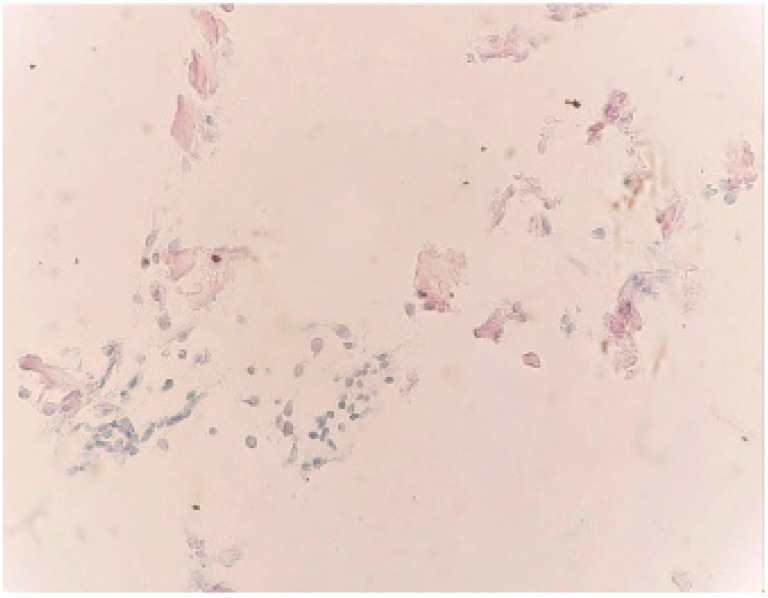
PAS-negative reaction of the unstructured calcium depositions (×40).

We also obtained a PAS (periodic acid Schiff)-negative reaction of the unstructured calcium depositions (×40) (Figure 8).

The common blood tests, performed during admission, were in the normal range.

The endocrinological evaluation, in the follicular phase, revealed: progesterone—0.39 ng/mL (0.05–0.89 ng/mL); estradiol—52 pg/mL (12.4–341.5 pg/mL); follicle-stimulating hormone (FSH)—8.56 mUI/mL (3.5–12.5 mIU/mL); luteinizing hormone (LH)—4.2 mIU/mL (1.9–14.6 mIU/mL); anti-mullerian hormone (AMH)—2.12 ng/mL (0.8–8.18 ng/mL); dehydroepiandrosterone sulfate (DHEA-S)—194 mg/dL (98.8–340 mg/dL), insulin-like growth factor 1 (IGF 1)—149 ng/mL (109–271 ng/mL), thyroid-stimulating hormone (TSH)—2.37 mIU/mL (0.27–4.2 mIU/mL) and cortisol—224 nmol/L (172–497 nmol/L).

Supplementary tests, for the possible explanation of abnormal calcifications, showed: calcium—8.9 mg/dL (8.6–10 mg/dL); phosphate—3.1 mg/dL (2.5–4.5 mg/dL) and vitamin D—42 ng/mL (20–50 ng/mL).

The semen analysis of her partner was normal.

The patient presented for transvaginal ultrasound control following the first menstruation and at 3 and 6 months after hysteroscopy. The exams revealed everything to be normal. She continued to have an annual regular gynecological/ultrasound examination.

Three years later, she returned with a positive pregnancy test. During this period, she sought out no investigations or treatment for infertility; she simply waited to conceive. 

Even if there was a desire for conception, the couple had not begun any infertility work-up for the last three years. Their reason, when asked, was their young age.

**Figure 9:**
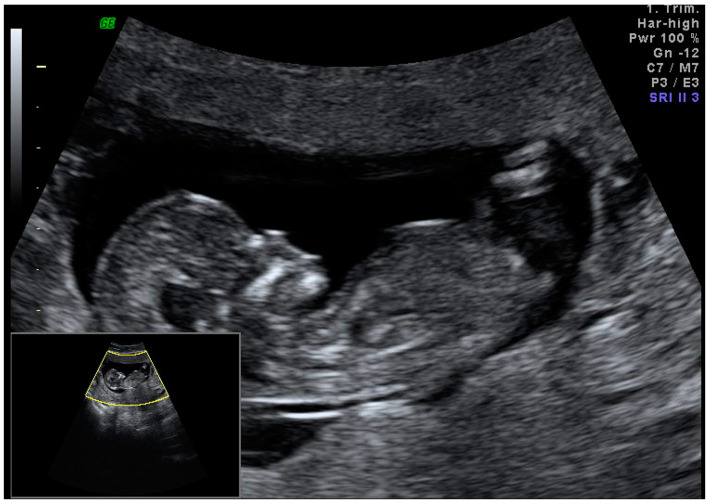
An ultrasound sagittal view of the fetus of 13 weeks and 2 days.

The ultrasound exam revealed a normal fetus, with a CRL of 73 mm, corresponding to a GA(LMP) of 13 weeks and 2 days (Figure 9).

The pregnancy was carefully, both clinically and with ultrasound, monitored, and it was uneventful until term. 

The patient decided for a cesarean section, because of her long period of infertility and intrauterine maneuver. She insisted on this and maintained her decision, after a thorough counseling. Finally, she delivered, at 38 weeks by C–section, a healthy boy weighing 3.280 g.

During surgery, no abnormal placenta adherence was observed. Furthermore, the placental histopathological exam was normal.

## 2. Discussion

In the majority of cases (72.9% of presentations), endometrial osseous metaplasia is an unexpected finding during an infertility investigation [11]. This entity is estimated to have an incidence of around 0.02% among infertile women [12].

Our patient complained, besides infertility, of menorrhagia. In the literature, EAO can be associated with a plethora of symptoms, from mild to severe ones. Khan et al. [7] found that the anomaly was asymptomatic in 5.6% of cases. The symptomatic patients revealed infertility (56.2%), irregular bleeding (19.8%), vaginal discharge (6.4%), dysmenorrhea (2.6%), dyspareunia (1.1%), pelvic pain (7.9%) and recurrent pregnancy loss (0.4%). In another communication, Parente et al. [9] reported that the most frequent complaint was menorrhagia (50%) followed by infertility (43%).

Over 90% of EOM described occurred after an abortion, either spontaneous, on demand or therapeutic [1]. For this reason, initially, it was considered that calcified tissue originates from a previous abortion [13]. But Cayuela et al. [14] demonstrated, after a comparative DNA analysis of bone fragments from a lesion and surrounding endometrium, that the tissue is of maternal origin. Later studies confirmed this finding and now it is generally accepted that the remaining embryonic/fetal tissue induces chronic endometritis. This phenomenon is responsible for the release of proinflammatory compounds, especially cytokines, as tumor necrosis factor alpha (TNFα) [1,6,7] and free superoxide radicals (secondary to the decreased expression of superoxide dismutase) [15]. These compounds can induce the metaplasia of endometrial (pluripotential) stromal cells to form osteoblast [16,17]. However, why only an extremely small number of abortions are followed by EOA remains unknown. 

In our case, however, there was no obstetrical history.

Wani et al. [1] reported on three patients with calcific endometritis and without a previous abortion. But, this entity, in contrast with EOM, exhibited histopathology characterized by an abundant of inflammatory cell infiltration and interspersed calcific foci. 

Furthermore, dystrophic calcifications can be secondary to genital tuberculosis [1,4,18], either in multi- or nulligravida patients.

The presence of the leukocyte infiltrate, in our case, raised questions about the presence of endometritis, but there was only weak leukocytic infiltration and CD 138 immunostaining additionally disproved this aspect. 

Furthermore, there was no specific cellularity for Koch bacillus infection.

PAS staining was used to rule out the origin of those deposits. The concretions could arise from hyaline bodies or psammomatous bodies. It was also able to exclude an infectious (especially fungal) etiology of endometrial calcification.

Some reports tried to correlate EOM with different metabolic disorders: hypercalcemia, hyperthyroidism, hyperphosphatemia or hypervitaminosis D, but the results were not sustained [9,19]. In any case, all these were normal for our patient.

In conclusion, we were unable to link the occurrence of this abnormality to any cause as her personal history and all laboratory tests, including endocrinological, immunological or electrolytical analysis (including calcium, phosphorus and vitamin D), were normal.

The first step in EOM diagnosis remains ultrasound due to its availability, wider acceptance and being a non-invasive procedure [1]. 

Hysteroscopy, followed by pathology, establish the final diagnostic. Due to its rarity, this entity is estimated to be present in only 0.15% of all referred hysteroscopies [9].

The per hysteroscopic resection of the endometrial bone corrected the menorrhagia and a spontaneous uneventful pregnancy occurred. 

Very probably, the infertility was the result of EOM. This conclusion was supported by the lack of any other abnormal finding during clinical and laboratory investigations and by the successful pregnancy after the extraction of the calcification.

Our finding is concordant with the literature, where the majority of authors reported on fertility recovering after hysteroscopy in EOM cases [1,6,7]. However, it should be stressed that a rate as high as 36% of spontaneous miscarriages can follow these procedures [7].

The mechanism involved in the infertility induction remains, however, unknown. It was proposed that the reactive endometritis, induced by the “new” bone, may interfere with the blastocyst implantation [20]. Another theory suggests that the bone tissue acts as an intrauterine contraceptive dispositive [21]. 

Finally, the unicity of the case presented occurs from its completely unknown etiology, especially in reference to the complete absence of obstetrical, but also endocrinological, infectious/inflammatory history, and despite a meticulous and complex investigation.

Also, the detailed histopathologic analysis of the calcific lesion should be noted.

## 3. Conclusions

We presented a rare case of endometrial osseous metaplasia. Unlike most cases of calcifications found in the endometrium, which have a history of abortion or chronic inflammation, our patient presented with neither.

The etiology of this abnormality remains unclear.

Very probably, there was a causal relation between its presence and infertility.

The diagnosis of endometrial osseous metaplasia is not an easy task, involving ultrasound, hysteroscopy and pathology. Regarding the treatment, hysteroscopic resection is the first choice for resolving this abnormality, with restored fertility in most of cases.

## Data Availability

Not applicable.
